# Insight into the Relationship of Spray-Drying Conditions with the Physicochemical and Gelation Properties of Egg White Protein

**DOI:** 10.3390/foods14091556

**Published:** 2025-04-29

**Authors:** Yuying Hu, Yan Hu, Huiyi Wu, Luyang Bao, Xin Shi, Can Wu, Bing Cui, Hongshan Liang, Bin Zhou

**Affiliations:** 1 Key Laboratory of Fermentation Engineering (Ministry of Education), Cooperative Innovation Center of Industrial Fermentation (Ministry of Education & Hubei Province), School of Life and Health Sciences, Hubei University of Technology, Wuhan 430068, China; 2 College of Food Science and Technology, Huazhong Agricultural University, Wuhan 430070, China

**Keywords:** egg white protein, spray-drying, gel, rheological property

## Abstract

This study aimed to provide systematic insight into the relationship between spray conditions and the physicochemical and gelation properties of egg white protein (EWP). Specifically, the effects of two key factors, the inlet temperature and flow rate, on the physicochemical and structural properties of EWP were determined. The analysis revealed that as the spray-drying temperature increased, more hydrophobic groups in EWP were exposed and prone to aggregate. Furthermore, the physicochemical and rheological properties and microstructure of egg white protein gel (EWPG) were determined. The results indicate that under a relatively high inlet temperature and a low flow rate, the hardness, springing, and water-holding capacity of the produced gel were improved. Excessively high temperatures were detrimental to pre-aggregate formation and the development of a homogeneous network. The rheological results demonstrate that the EWPG exhibited a weak frequency dependence and elastic-dominant gel characteristics. Further analysis indicated that the inlet temperature significantly influenced the nonlinear response of the EWPG, with the strongest higher-order viscous nonlinear properties observed at 140 °C. The microstructure suggested that at 140 °C, the EWPG achieved a minimum porosity of 50.07% and a maximum fractal dimension (D_f_) of 2.745, where a uniform network structure was generated. This study demonstrated that relatively high temperatures and low flow rates in the spray-drying process were advantageous for producing egg white protein gel with desirable characteristics, which has potential for the actual application of egg-based food products.

## 1. Introduction

As the main component of raw eggs, egg whites comprise 13%~15% egg white protein (EWP), containing eight kinds of essential amino acids for humans. Moreover, EWP exhibits many excellent properties, including gelation, foaming, and emulsification, and is widely used in food products, such as meat products, cakes, and ice cream [1]. Egg white protein is the dried egg product obtained from fresh egg white, which is produced by multiple processes, including cleaning, sterilizing, separating, filtering, homogenizing, and drying. In the production process of egg white protein, drying is a key procedure linked to the quality of egg products. It is reported that many kinds of drying methods are used in actual industry, for instance, hot air-drying, spray-drying, vacuum freeze-drying, microwave-drying, far-infrared-drying, and so on [2,3].

Among them, spray-drying is an effective approach to produce egg white protein, where the drying process is quick enough to protect the protein from severe denaturation. During this process, the liquids containing dissolved or suspended solids are converted into dried powders [4]. Egg white protein is generally produced at high temperatures, which leads to changes in the egg constituents and results in different functional properties of the egg white protein. For instance, the inlet temperature of spray-drying (165~190 °C) leads to a reduction in the emulsifying and gelling ability of egg white protein [5]. Some studies found that as the inlet temperature of spray-drying ranged from 110 °C to 125 °C, the water-holding capacity of the produced egg white protein gel (EWPG) was considerably improved [6]. Meanwhile, the flow rate is another key factor affecting the properties of the protein produced during the spray-drying process. With a declining flow rate, the dimension of droplets produced in the airflow atomizer would decline, resulting in a long stay of the material in the spray-drying process [7]. Hence, during the spray-drying process of egg white protein, both the inlet temperature and flow rate are the critical factors that significantly influence the physicochemical properties of the egg white protein, as well as the functional properties of the produced gel.

Indeed, rheology has been widely adopted in the evaluation of gel systems to determine viscoelasticity, including temperature sweep, frequency scan, strain scan, and creep–recovery. In the small-amplitude oscillatory shear (SAOS) method, the sample is subjected to small-amplitude oscillatory deformation to assess its viscoelastic properties, suggesting that SAOS measurement could test its viscosity and elasticity without disturbing the gel structure [8]. However, the real food system is generally subjected to large deformation during production and chewing. Hence, large-amplitude oscillatory shear (LAOS) is a more effective method to provide information on the physicochemical properties of food products in real applications. Currently, there is much literature reporting the non-linear rheological properties of gels over a wide range of strains and frequencies by LAOS. Ma et al. [9] investigated the effect of the carboxymethyl flaxseed gum concentration on the non-linear rheological properties of the mixture suspension under LAOS. Also, the research by Dai et al. [10] observed the strain-hardening behavior of a desalted duck egg white/gelatin mixture under LAOS. However, there is less research focused on the LAOS of egg white protein gel, and the information about the nonlinear rheological behavior of egg white protein gel under spray-drying is limited, which restricts the development of actual egg-based products.

Therefore, this study aimed to examine the effects of the inlet temperature and flow rate of spray-drying on the physicochemical properties of egg white protein, including solubility, protein structure, and selective solubility, and the structure and rheology of egg white protein gel were determined, which is meaningful in the exploration of the potential application of eggs. In particular, the rheological properties under SAOS and LAOS of egg white gel under different processing conditions were compared to provide in-depth insight into the relationship with the structure and properties of the egg white protein gel. This study would be useful in optimizing the processing conditions of spray-drying to obtain products with desired properties and provide a useful, practical basis for egg manufacturing, which would expand the application of egg-based products.

## 2. Materials and Methods

### 2.1. Materials

Fresh eggs (Shendi Agriculture Branch Trade, Jingshan, China) were purchased. All chemical reagents (Sinopharm Chemical Reagent Co., Ltd., Shanghai, China) were of analytical grade. Ultrapure water was adopted for sample preparation in all experiments.

### 2.2. Process of Spray-Drying

Spray-drying (YC-510 Spray Dryer, Yacheng Instrument and Equipment Co., Ltd., Shanghai, China) was applied to produce EWP with a spray pressure of 0.2 MPa, and the inlet temperatures were set to 100 °C, 120 °C, 140 °C, and 160 °C, and the flow rates were set to 720, 900, 1080, and 1260 mL/h, respectively.

### 2.3. Production of Egg White Protein and Gel

Fresh eggs without cracks were selected, washed, and dried, and then the egg whites were separated from the yolks. The solids content of the egg white was 11.28%, which was determined by the Bradford method. The egg white was stirred at 200 r/min for 5 h and passed through an 80-mesh sieve, and, subsequently, the pH was adjusted to 9.0 using a citric acid solution (40%, *w*/*w*). The processed egg white was spray-dried according to the conditions in the following Table 1. The spray-dried egg white protein (EWP) was reconstituted into a 10% (*w*/*w*) EWP solution. The solution was filled into a food-grade plastic enteric coating (40 mm in diameter), heated at 90 °C for 30 min, and then immediately immersed in ice water for cooling to produce egg white protein gel (EWPG), which was refrigerated at 4 °C overnight.

### 2.4. Physicochemical Measuring of EWP

The solubility of EWP was examined according to the method of Li et al. [11], with minor modifications. In general, EWP (2 g) was dispersed in deionized water (20 mL), and then centrifuged (9000 r/min; 10 min). Finally, the precipitate was obtained and dried. This process was repeated until the precipitation was weighed to a constant value and computed as follows:

(1) $$Solubility/(\frac{g}{100\;g})=100-\frac{\left(m_{2}-m_{1}\right)\times 100}{(1-B)\times m}$$

where *m* represents the mass of the original sample (g); *m*_1_ represents the mass of the centrifuge tube (g); *m*_2_ represents the mass (g) of the centrifuge tube and insoluble matter after drying; and B represents the moisture content of the sample (g/g).

Prior to the examination of the pH test, the solution was diluted to reach a solid content of 10% and then stirred (450 rpm) for 1 h, and the pH of the EWP solution was determined by a pH meter (FE-28, Mettler Toledo, Shanghai, China).

The turbidity was measured with a UV–visible spectrophotometer (UV2550, Shimadzu, Kyoto, Japan) at a wavelength of 600 nm, and each sample was dispersed in deionized water to achieve a protein solution of 10 mg/mL.

The particle size characteristics were determined within a Zetasizer Nano instrument (Nano ZS, Malvern Instruments Ltd., Malvern, UK). In this measurement, the indices of the egg white protein and water phase were 1.45 and 1.33 [12], respectively.

### 2.5. Structural Characterization of EWP

The surface hydrophobicity of EWP was examined according to the method of Dehghannya et al. [13], with slight modifications. The surface hydrophobicity was determined by adding an 8 mM 1-anilinonaphthalene-8-sulfonate (ANS) solution to the protein solutions (0.10–0.50 mg/mL in 50 mM PBS; pH 7.2) at a volumetric ratio of 1:40 (ANS–protein solution). The fluorescence intensity was measured using a spectrofluorometer (F-4500, Hitachi High-Technologies Co., Ltd., Tokyo, Japan) with a xenon lamp and a 1.0 cm quartz cuvette. The measurements were conducted at excitation/emission wavelengths of 390/470 nm, with both slit widths set to 5 nm. The surface hydrophobicity was determined by the fluorescence intensity and protein concentration.

Following Wang’s method, with minor modifications [14], EWP was dissolved in Tris–Gly buffer (0.1 M Tris, 0.1 M glycine, and 4 mM EDTA; pH 8.0) to prepare a 30 mg/mL protein solution. This solution was reacted with 50 μL of Ellman’s reagent (4 mg/mL DTNB dissolved in Tris–Gly buffer) under dark conditions at 25 °C for 1 h. After centrifugation at 10,000 r/min for 10 min, the absorbance of the supernatant was measured at 412 nm. For the total sulfhydryl content (SH_T_) determination, EWP was dissolved in Tris–Gly buffer (0.1 M Tris, 0.1 M glycine, 4 mM EDTA, 0.5% SDS, and 8 M urea; pH 8.0) to prepare a 30 mg/mL protein solution, followed by the same fluorometric analysis as for free sulfhydryl groups (SH_F_). All measurements were performed in triplicate. The total sulfhydryl (SH_T_), free sulfhydryl (SH_F_), and disulfide bonds (SS) were calculated by the following formulas:

(2) $$SH\left(\mu mol/g\right)=73.53\times A_{412}\times D/C$$

(3) $$SS\left(\mu mol/g\right)=({SH}_{T}-{SH}_{F})/2$$

where A_412_ is the absorbance (at 412 nm), C is the protein concentration, and D is the dilution fold.

### 2.6. Gel Properties Measurement of EWPG

Following Domian’s method, with minor modifications [15], the gel properties of EWPG were tested using a texture analyzer (TA. XT Plus, Stable Microsystems, Surrey, UK) with a P/36R probe and carried out with the following parameters: the pre-test rate (5.0 mm/s), test rate (2.0 mm/s), post-test rate (5.0 mm/s), and compression level (50%).

Following Fang’s method, with minor modifications [16], the water-holding capacity (WHC) was determined by transferring 2.0 g of EWPG into a 50 mL filter paper-lined centrifuge tube, followed by centrifugation at 8000 r/min for 10 min. The WHC values were subsequently calculated using the established formula:

(4) $$WHC\left(\%\right)=\frac{m_{2}}{m_{1}}\times 100\%$$

where *m*_1_ was the weight of the EWPG before centrifugation, and *m*_2_ was the precipitate mass obtained after centrifugation.

Following Wang’s method with minor modifications [14], selective solubility was determined using the method described below: 2 g of EWPG was dispersed into 18 mL of solvents: S0 (0.05 mol/L NaCl); S1 (0.6 mol/L NaCl); S2 (0.6 mol/L NaCl + 1.5 mol/L urea); S3 (0.6 mol/L NaCl + 8 mol/L urea); and S4 (0.6 mol/L NaCl + 8 mol/L urea + 0.5 mol/L β-ME). The concentration of the supernatant at 595 nm was determined using bovine serum protein as the standard, and the corresponding content of the sample was calculated from the standard curve.

### 2.7. Rheological Test of EWPG

The rheological properties of the EWPG were tested using an Anton Para MCR 92 rheometer (Anton Para, Graz, Austria) equipped with a parallel plate (diameter: 50 mm; gap: 1 mm). The test pattern was carried out as follows:

Frequency sweeps were carried out in the 0.01–10 Hz range at a small strain amplitude of 1.0% (within the LVR) [17], and both the storage modulus (G′) and loss modulus (G″) were recorded. Then, the resulting data were fitted to a power-law model to determine the frequency dependence of G′ and G″.

(5) $$G^{\prime }={K^{\prime }\left(\omega \right)}^{n^{\prime }},G^{\prime \prime }=K^{\prime \prime }{\left(\omega \right)}^{n^{\prime \prime }}$$

where ω is the frequency in rad/s, n′ and n″ are power-law exponents, and K′ and K″ are constants.

The temperature sweep was carried out as follows: the EWP solution was heated to 90 °C at a constant rate of 5 °C/min and maintained for 30 min and then cooled down at a constant rate of 5 °C/min. Also, the storage modulus (G′) and loss modulus (G″) in the temperature sweep were obtained. The immobilization scan frequency was 1 Hz at a strain of 1.0% [10].

The creep–recovery test was performed at 150 Pa within the linear viscoelastic region, and the strain in response to the constant stress was measured over 300 s. Within the recovery range, the applied stress was suddenly removed, and the sample recovered within 600 s, where strain γ (%) was recorded. Then, the creep curve was fitted by the Burgers model. The flexibility (compliance, J) was the strain per unit stress and calculated using the following expression [18]:

(6) $$J_{\left(t\right)}=J_{0}+J_{1}\left(1-EXP(-t/\lambda \right))+t/\eta_{0}$$

where J_(t)_ is the overall compliance at any time t, J_0_ is the instantaneous elastic compliance, J_1_ is the retarded elastic compliance, λ is the retardation time, and η_0_ indicates zero shear viscosity.

For large-amplitude oscillatory shear, the intra-cycle stress data were analyzed using the MITlaos software (MITlaos Ver. 2.1 Beta), which provided the intra-cycle stress decomposition from raw waveforms, and the Chebyshev stress decomposition was analyzed [19].

### 2.8. Microstructure of EWPG

The EWPG was cut into squares of 0.3 cm (side length) and immersed in glutaraldehyde solution (2.5%, *v*/*v*) overnight for fixation. Then, it was rinsed three times with 0.1 M PBS in advance. Following freeze drying, the EWPG samples were sputter-coated with gold prior to microscopic observation. Their cross-sectional morphology was examined using a scanning electron microscope (JSM 6390LV, JEOL Ltd., Tokyo, Japan) operating at a 15 kV accelerating voltage.

Porosity analysis: scanning electron microscopy images of the EWPG at a magnification of 500 were selected for analysis. The porosity of the EWPG was analyzed by the ImageJ software (Ver. 1.54f), and the porosity was calculated by referring to the following equation:

(7) $$Porosity\left(\%\right)=\left(Pore\;area/Total\;image\;area\right)\times 100\%$$

Moreover, the SEM images captured at a 500× magnification were converted into 8-bit binary images using the ImageJ software (National Institutes of Health, Bethesda, MD, USA) and subjected to threshold processing. The fractal dimension (D_f_) was determined via the box-counting method using ImageJ’s Fractal Box Counter plugin, which quantifies structural complexity by analyzing pixel density variations across multiple scales [17].

### 2.9. Statistical Analysis

At least three replicates were performed for all measurements. Statistical differences (*p* < 0.05) were analyzed by one-way analysis of variance (ANOVA) using the SPSS 24.0 software (IBM, Chicago, IL, USA), and the data were expressed as means and standard deviations. The Origin 2024 software (Origin Lab Corporation, Northampton, MA, USA) was used for the analysis and graphing.

## 3. Results and Discussion

### 3.1. Physicochemical Properties of EWP

As shown in Table 2, the effects of the inlet temperature and flow rate on the solubility and pH of EWP are displayed. It was found that the solubility of EWP significantly declined when the inlet temperature was higher than 140 °C. This may be due to the high temperature leading to protein denaturation and the exposed hydrophobic groups obstructing the diffusion of protein molecules into the water, resulting in a reduction in solubility. The egg white powder treated at 100 °C exhibited a higher moisture content compared with the other samples, which may induce water migration during storage. This phenomenon could potentially promote the intermolecular aggregation of unfolded protein chains through enhanced molecular mobility and hydrophobic interactions, ultimately leading to a reduced solubility of the powder [20]. After spray-drying, the pH of the EWP significantly increased (from 9.0 to 10.26~10.55). This change generally contributed to the exposure of the lateral chains of the basic amino acid residues, such as arginine and lysine, in the protein molecules. As the inlet temperature increased, the degree of exposure to alkaline amino acid residues in the egg white proteins progressively increased. Notably, the ε-amino groups of lysine (pKa ≈ 10.5) and the guanidino groups of arginine (pKa ≈ 12.5) became more accessible in the denatured protein matrix. In concentrated protein systems without a buffering capacity, these exposed basic residues preferentially bind to protons, thereby significantly increasing the solution pH through equilibrium displacement. Similar phenomena have been documented in previous studies by Katekhong et al. [20] and Ayadi et al. [6].

**Figure 1:**
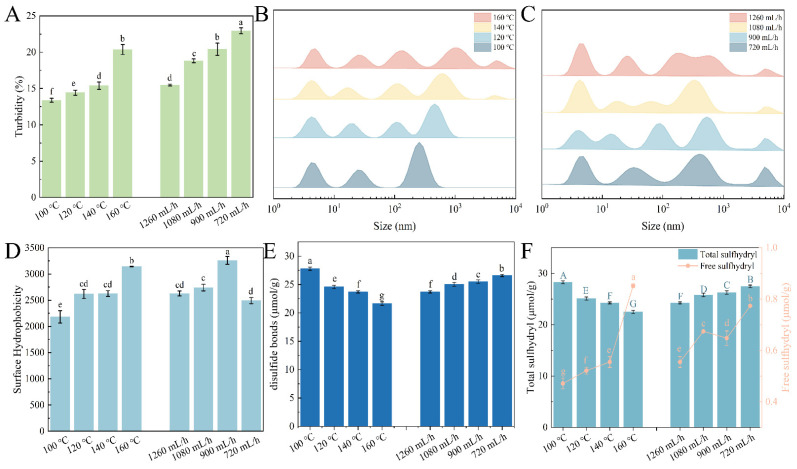
Relationship between inlet temperature and flow rate with physicochemical properties of EWP: (**A**) turbidity; (**B**,**C**) particle size distribution; (**D**) surface hydrophobicity; (**E**) disulfide bonds; and (**F**) total and free sulfhydryl content. The different lower-case letters in (**A**,**D**,**E**) and uppercase letters in (**F**) respectively indicate the significant differences (*p* < 0.05) of free and total sulfhydryl content among the samples.

The turbidity and particle size are presented in Figure 1A–C, which reflect the degree of EWP aggregation after spray-drying. While increasing the inlet temperature (100~160 °C), the turbidity rose from 13.37% to 20.35%, which indicated an increased aggregation degree of the EWP. Meanwhile, after spray-drying, the particle size distribution (PSD) of all EWP samples was multimodal, suggesting that there were protein monomers, oligomers, and larger protein aggregates. And with the increasing inlet temperature and decreasing flow rate, the PSD of the EWP shifted to a large size, which was attributed to the generation of larger protein aggregates. A similar result was documented in other literature by Yang et al. [21], where it was observed that the heating treatment within spray-drying induced the formation of soluble protein aggregates of pea protein isolates.

### 3.2. Structural Changes of EWP

The structural changes of the EWP during spray-drying are depicted in Figure 1D. With the increasing inlet temperature, the surface hydrophobicity of the EWP significantly rose from 2181 to 3142. This phenomenon indicated that there was more exposure of hydrophobic regions as the inlet temperature increased, leading to a higher degree of protein denaturation. This was in line with the findings in Section 3.1. In addition, it was observed that the surface hydrophobicity was at a maximum value of 3259 at the flow rate of 900 mL/h. This may be ascribed to the low flow rate, which led to prolonging the residence time of particles in the drying chamber and induced more exposure of hydrophobic groups in the protein molecules.

The content of total and free sulfhydryl, as well as disulfide bonds, is given in Figure 1E,F. As shown, when the inlet temperature rose from 100 °C to 160 °C, the total sulfhydryl significantly decreased from 28.26 to 22.49 μmol/g, and the disulfide bonds reduced from 27.79 to 21.63 μmol/g, which was related to the breaking of peptide bonds [22]. The free sulfhydryl slightly increased from 0.47 to 0.85 μmol/g, suggesting that the unfolding of the protein structure was induced at high temperatures, and the internal structure was exposed, resulting in a higher content of free sulfhydryl at 160 °C. Meanwhile, all contents presented an increasing tendency with a reduced flow rate. In terms of the EWP at 720 mL/h, a lower flow rate indicated a reduction in moisture in the drying chamber, which led to a higher outlet temperature [23]. And the reduction in the flow rate led to a long stay in the drying chamber, which was beneficial for the formation of disulfide bonds. This was consistent with the results [24].

### 3.3. Gel Properties of EWPG

**Figure 2:**
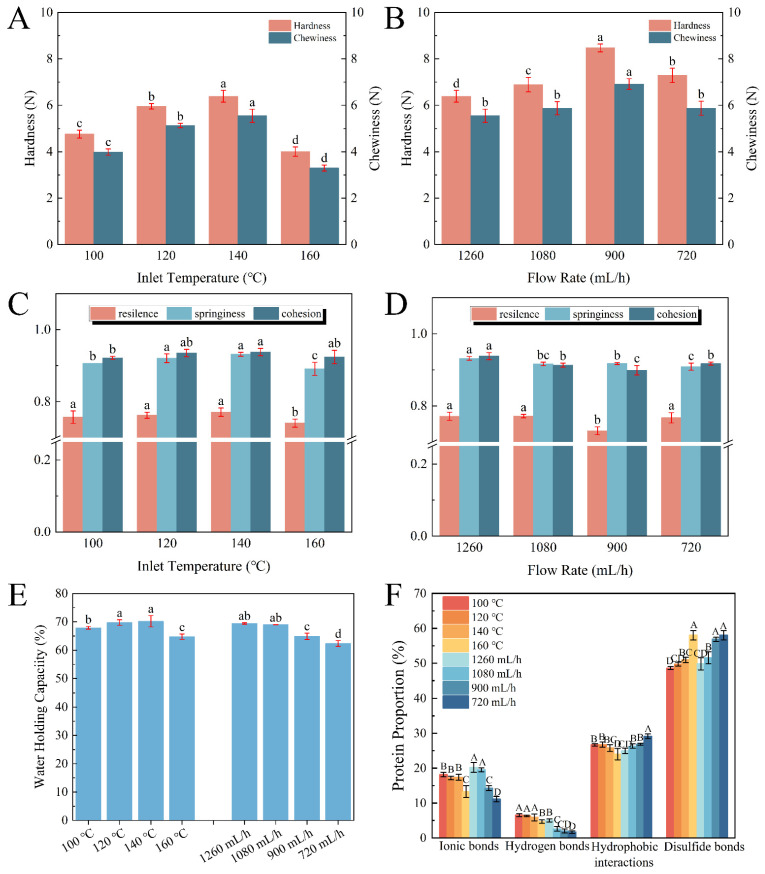
Relationship between inlet temperature and flow rate with gel properties of EWPG: (**A**,**B**) hardness and chewiness; (**C**,**D**) resilience, springiness, and cohesion; (**E**) water-holding capacity; and (**F**) intermolecular force. The different lower-case letters in (**A**–**E**) and uppercase letters in (**F**) respectively indicate the significant differences (*p* < 0.05) among the samples.

In order to provide further insight into the relationship of the spray-drying conditions with the produced protein, the gel properties of the egg white protein gel (EWPG) were assessed by some measurements, such as TPA, water-holding capacity, selective solubility, and so on. Spray-drying induced the partial denaturation of the EWP and the formation of primary aggregates, which facilitated the formation of a gel network structure. As shown in Figure 2A–D, the highest hardness (6.38 N) and chewiness (5.55 N) were observed in the sample treated at 140 °C. And with the declining flow rate, both hardness and chewiness displayed an increasing tendency. The highest hardness (8.43 N) and chewiness (6.92 N) appeared at a flow rate of 900 mL/h. All resilience values were higher than 75%, suggesting a great ability to rapidly revert deformation after compression. And when the inlet temperature exceeded 140 °C, the springiness of the EWPG significantly reduced to 87.84%. This phenomenon was linked to the formation of insoluble aggregates of proteins, leading to the generation of an inhomogeneous gel network. The cohesion was highest (93.79%) at 1260 mL/h. As reported, hardness and chewiness are generally dominant in the cross-linked degrees of protein molecules [14], and a higher inlet temperature and a lower flow rate were advantageous for the denaturation of the protein, and then there were ample association and aggregation reactions to form a soluble egg white protein network with a stable three-dimensional structure. The results suggest that in the process of spray-drying, a high inlet temperature and a low flow rate were beneficial in inhibiting the deterioration of the EWPG.

As shown in Figure 2E, as the inlet temperature was higher than 140 °C, the water-holding capacity (WHC) declined from 70.2% to 64.79%. This was due to the WHC being linked to the microstructure of the heat-induced gel, where a gel with a loose pore structure generally displays a low WHC [25]. Higher temperatures induced a larger denaturation degree of the egg protein, which was adverse to the formation of gel. Moreover, the WHC of the EWPG was reduced with a decreasing flow rate. The probable reason was that the low flow rate led to a longer stay in the spray-drying process, which contributed to the excessive aggregation of the EWP, resulting in a low WHC [26]. Hence, a proper inlet temperature and flow rate were beneficial to produce EWPG with a high WHC.

As shown in Figure 2F, the intermolecular force in the protein-based gel was determined, and the dominant forces in the EWPG were disulfide bonds and hydrophobic interactions, which accounted for almost 75%, followed by ionic bonds (about 20%), while hydrogen bonds accounted for less than 10%. In general, disulfide bonds were the main force that maintained the structure of heat-induced EWPG. The hydrophobic interactions presented a slightly decreasing trend with the increasing inlet temperature and increased significantly with the decreasing flow rate. As reported, moderate heat treatment was advantageous to the unfolding of the EWP molecules, while extremely high temperatures induced the generation of insoluble aggregates, which was adverse for gelation [27]. When the inlet temperature was higher than 160 °C, the proportion of ionic bonds apparently reduced with the increasing temperature. Also, with a decline in the flow rate in spray-drying, this proportion of ionic bonds significantly declined. This result may be ascribed to the excessive aggregation of protein cross-links, which hindered the interaction between ions and protein molecules [28]. There were numerous sulfhydryl groups and disulfide bonds in the EWP (Figure 1E,F). Cross-linking between proteins via disulfide bonds promoted the gelation of the EWP [29]. It was shown that the proportion of disulfide bonds pronouncedly increased with the increasing degree of thermal treatment.

### 3.4. Rheological Properties of EWPG

**Figure 3:**
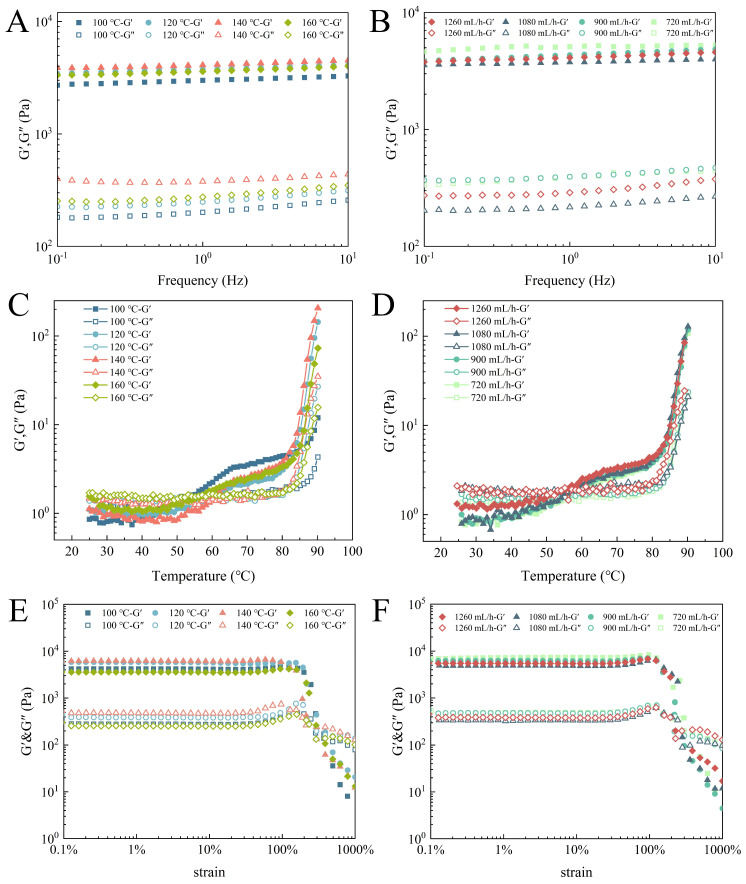
Rheological properties of EWPG: (**A**,**B**) frequency sweep; (**C**,**D**) temperature sweep; and (**E**,**F**) strain sweep.

The frequency sweep of the EWPG is displayed in Figure 3A,B. In the range of 0.1~10 Hz, the value of G′ was always larger than G″, indicating that the EWPG displayed a continuous network structure and exhibited the features of a solid gel. The G′ of EWPG reached the maximum value at an inlet temperature of 140 °C. Then, it declined with the increasing temperature, which may be attributed to the irreversible denaturation of the protein under a high inlet temperature. As exhibited in Table 3, the power-law indices of the EWPG ranged from 0.021 to 0.085, indicating that the EWPG displayed a weak frequency dependence, with excellent elasticity. It was found that with increasing inlet temperature and decreasing flow rate, both K′ and K″ were significantly enhanced, suggesting that a suitable inlet temperature and flow rate improved the pre-aggregation of the EWP, which was linked to the viscoelasticity of the gel [30]. The gelation curves of the EWPG during temperature sweeping are exhibited in Figure 3C,D. It was seen that all samples showed a typical sol–gel transition. G′ displayed two apparent increases at about 50 °C and 80 °C, and G″ was significantly enhanced at around 80 °C. This phenomenon was related to the denaturation temperature of ovalbumin and ovotransferrin in the EWP [31]. With the increasing inlet temperature, the gelation temperature (T_g_) values were 50.19 °C, 55.20 °C, 60.19 °C, and 58.19 °C, respectively. This result demonstrated that the process of spray-drying induced the denaturation of some parts of the EWP and led to decreased enthalpy required for protein denaturation, resulting in a reduction in the T_g_ [32]. Moreover, a previous study documented that the treatment of spray-drying induced EWP to be in a pre-aggregated state, and the presence of large soluble aggregates accelerated the EWPG gelation process. In this process, large aggregates were preliminarily precipitated and interacted to form a primary network; subsequently, small aggregate particles and soluble proteins bound to the network to produce the network structure [30]. And it was found that there were no significant effects on the T_g_ of the EWPG with different flow rates. Therefore, it could be concluded that the inlet temperature was the main factor affecting the T_g_ of the EWPG in the spray-drying process. The result of the strain sweep is exhibited in Figure 3E,F. In the range of small strains, the moduli did not change within the strain, suggesting that it was in the linear viscoelastic region [33]. Near the critical strain, both the G′ and G″ increased initially and decreased subsequently. This was defined as strong strain overshoot (LAOS type IV) [34]. This kind of gel network may be formed by some large aggregate clusters or hydrophobic groups [35]. In addition, pronounced strong strain overshooting behavior would be observed at higher frequencies [36]. Xia et al. [37] also found this phenomenon in soy protein gel, and they attributed it to the entanglement of protein aggregates.

**Figure 4:**
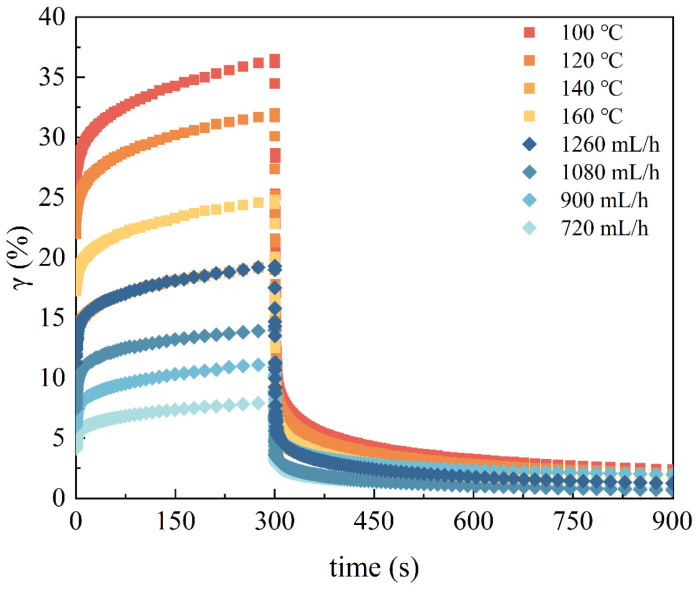
Creep–recovery curves of EWPG within different inlet temperatures and flow rates in the spray-drying process.

The creep–recovery curve of the EWPG is displayed in Figure 4. A constant stress (300 Pa) was applied to the EWPG, and deformation happened instantaneously (0~0.9 s). And the strain of the EWPG continuously increased over time. The maximum strain of the EWPG varied, where a larger deformation indicated it was softer and easier to stretch. In addition, it was found that the gel could not recover completely as the constant stress was removed, indicating that the viscous component dissipated the deformation energy [10]. The Burger model was adopted to fit the creep behavior of the EWPG, and all fitting coefficients (R^2^) were larger than 0.95, suggesting that this model fit the system of the EWPG well. Furthermore, the compliance at the end of creep was maximum compliance (J_max_), which represents the maximum resistance of a sample to strain and reflects the bond strength between the structural units of the sample [38]. As shown in Table 4, J_max_ presented a minimum value at 140 °C and gradually declined with the decreasing flow rate. And the trends of J_0_ and J_1_ were consistent with J_max_. The inlet temperature and flow rate were related to the sulfhydryl-binding site exposure of the egg white protein and led to more generation of disulfide bonds during pre-aggregation, resulting in an enhancement in the interactions between protein molecules. When the inlet temperature was too high, the excessive pre-aggregation was detrimental to the formation of a homogeneous network and then led to an increase in the maximum deformation of the gel [30]. As exhibited in Table 3, the delay time (λ) characterizes the response of a viscoelastic material to an instantaneously applied constant pressure [39], where a longer delay time suggests a slower elastic response. As the inlet temperature increased from 100 °C to 140 °C, the value of λ displayed a significant reduction. This result suggested a fast elastic response of the EWPG, which was beneficial for its recovery after deformation. The recovery (R%) of the entire system is expressed as the percentage of the reversible compliance to the maximum compliance [40]. The results indicate that the produced EWPG had good recoverability with high elasticity and high resistance to deformation.

**Figure 5:**
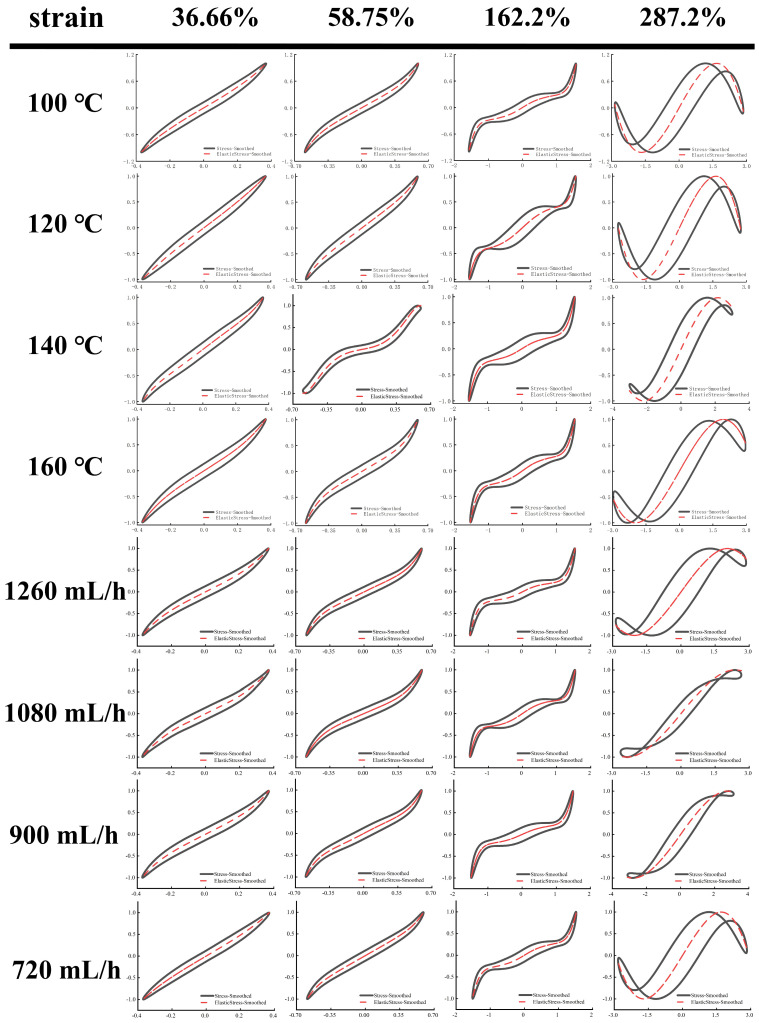
Elastic Lissajous–Bowditch curves of EWPG under different inlet temperatures and flow rates. Note: The black point represents total intracycle normalized stress, and the red line represents normalized stress decomposition. All instantaneous stress values are normalized relative to the maximum stress within the oscillation cycle.

**Figure 6:**
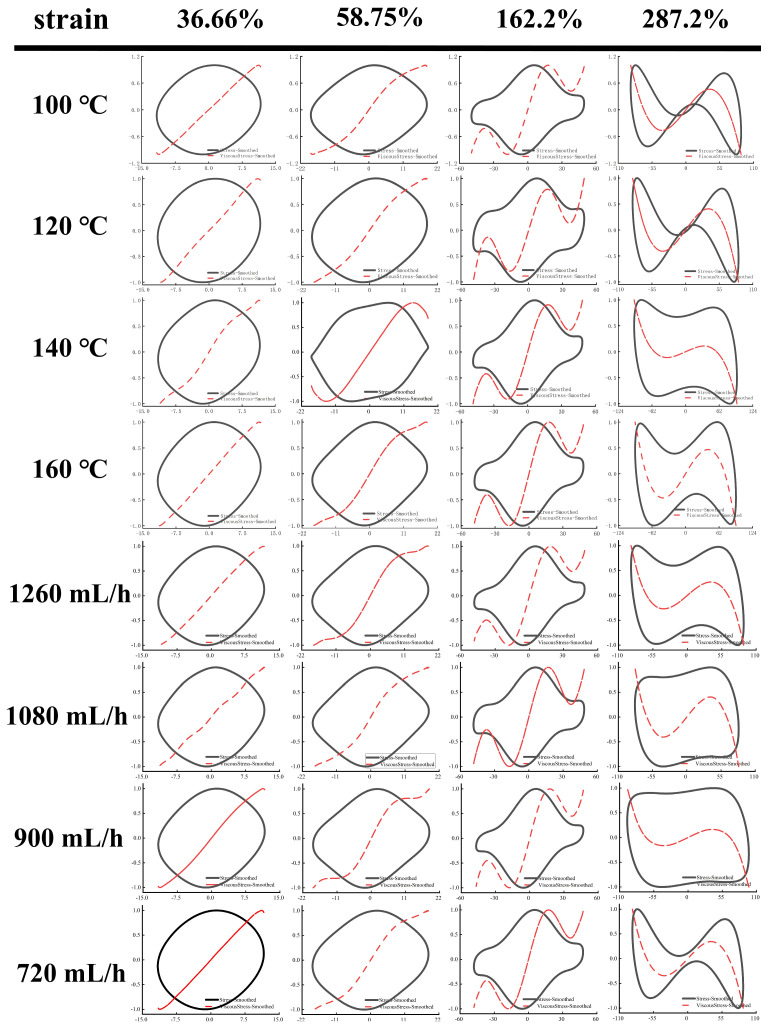
Viscous Lissajous–Bowditch curves of EWPG under different inlet temperatures and flow rates. Note: The black point represents total intracycle normalized stress, and the red line represents normalized stress decomposition. All instantaneous stress values are normalized relative to the maximum stress within the oscillation cycle.

As shown in Figure 5 and Figure 6, Lissajous–Bowditch (LB) curves were applied to analyze LAOS. As shown, when the material had a high elasticity, the elastic LB curve exhibited a straight line or a narrow ellipse shape, and the distortion degree of the curve was linked to the nonlinear response of the stresses [35]. In these elasticity curves, all EWPG samples presented a narrow elliptical shape at strain (<36.66%), and the decomposition stresses corresponded with the total stresses, suggesting that the EWPG was an elastic linear material. In these viscous LB curves, with increasing strain, the slope of the viscous stresses began to change, indicating the coexistence of an elastic linear with a viscous nonlinear contribution. In the strain range of 36~162%, the elastic component of stress achieved a maximum around the slope of γ = ±γ_0_, which exhibited intra-cycle strain stiffening. This finding documented that the inlet temperature had a significant effect on the intra-cycle strain stiffening. This behavior first appeared at 36.66% in the EWPG treated within 140 °C and 160 °C. In comparison, the elastic LB curve was more distorted at 140 °C, suggesting a larger contribution from elastic nonlinearity. In contrast, the viscous LB curve was more complicated. As the strain increased, the LB curve gradually transferred from a broad ellipse to a parallelogram or hexagon, and a multi-waveform appeared at a viscous stress component of 58.75%. It can be observed that the EWPG first exhibited intra-cyclic shear thickening near an instantaneous strain rate of zero, followed by intra-cyclic shear thinning. And finally, intra-cyclic shear thickening appeared again near an instantaneous strain rate of the maximum value. This result may have been caused by the generation of some hyper-entanglements in the network structure of the EWPG, where at a low instantaneous strain, these hyper-entanglements gradually yielded and collapsed instantaneously as the instantaneous strain increased. While the whole network was not damaged, shear thickening and shear thinning occurred successively in a cycle. Subsequently, the strain increased near the maximum value, and the whole network was close to yielding, and cyclic shear thickening occurred again [41]. A similar phenomenon was also observed in the research by Goudoulas and Germann [19]. When the strain amplitudes were larger than 162%, the LB curve became more complicated, and all curves in the elastic LB curve appeared to be secondary loops, corresponding to self-intersecting and strongly nonlinear viscous responses at large strain amplitudes. This self-intersection phenomenon was also investigated in previous research by Tong et al. [42], where high- and low-acyl gellan gum composite systems were determined. This may be due to the fact that the time scale of gel structure restructuring was shorter than that of oscillatory deformation and was related to the faster unloading of transient instantaneous elastic stresses than the accumulation of new elastic deformation [43].

**Figure 7:**
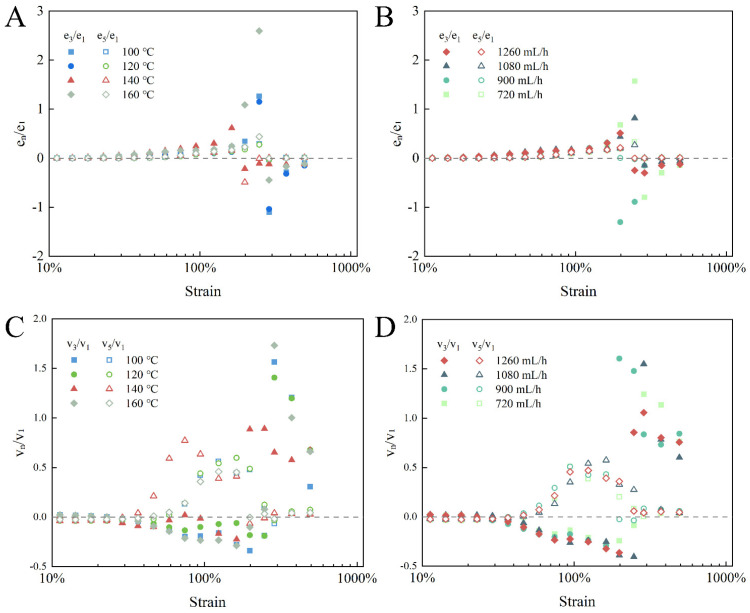
Chebyshev coefficient ratios calculated for EWPG: (**A**,**B**) elastic nonlinear measures; (**C**,**D**) viscous nonlinear measures.

A quantitative analysis of the inter-cycle nonlinearity degree could be determined by evaluating the Chebyshev coefficient ratios. As displayed in Figure 7, as the strain was not sufficient for yielding, the ratio of e_3_/e_1_ presented an increasing tendency, suggesting that before the network broke down, there was a continuously growing strain-stiffening response [16]. Before yielding, all e_3_/e_1_ values were well fitted, and the higher-order elasticity ratio (e_5_/e_1_) was not changed in the yield strain range; therefore, it could be demonstrated that the stiffening characteristic was driven by the third-order nonlinear harmonic [44]. At the yield point, the ratio of e_3_/e_1_ was less than zero and gradually approached zero, presenting a gradual weakening of the strain-softening response, indicating gel network fragmentation. The viscous Chebyshev coefficient ratios (v_3_/v_1_) were near zero in a linear state. However, as the strain amplitude increased from SAOS to LAOS, the ratio of v_3_/v_1_ was less than zero, while v_5_/v_1_ was larger than zero, indicating that the intro-cycle shear-thinning property of the EWPG was dominantly affected by higher-order nonlinear viscous harmonics [45]. These results demonstrate that the inlet temperature had a significant effect on the higher-order nonlinear response of the EWPG, where the shortest linear viscoelastic zone and strongest higher-order viscous nonlinear properties appeared in the EWPG at 140 °C, and its elastic nonlinear response was weaker.

### 3.5. Microstructural Features of EWPG

**Figure 8:**
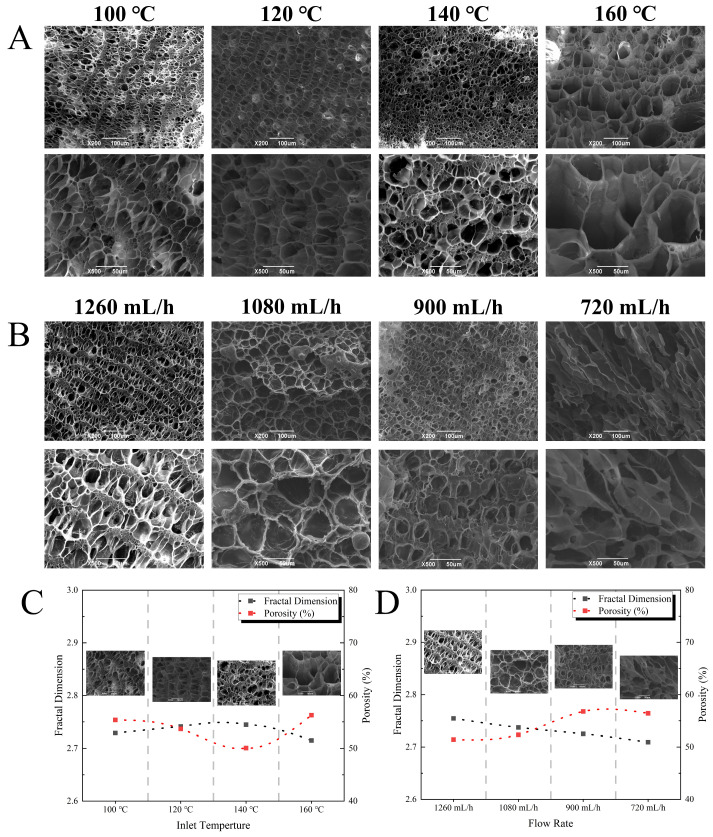
(**A**,**B**) Scanning electron microscopy; (**C**,**D**) porosity and fractal dimension of EWPG under different inlet temperatures and flow rates.

The microstructure of the EWPG was observed using scanning electron microscopy (JSM 6390LV, JEOL Ltd., Tokyo, Japan). As shown in Figure 8, when the inlet temperature was lower than 160 °C, the EWPG exhibited a dense gel network structure, and the pores were laminar-like. When the temperature was higher than 160 °C, some larger pores were exhibited in the EWPG. As for the flow rate, the microstructure of the EWPG at 720 mL/h exhibited a fragmented sheet structure, while it presented a more regular network structure at a flow rate of 1080 mL/h. Then, the SEM image was examined using ImageJ to analyze the porosity and fractal dimension (D_f_) of the network structure. The porosity represented the size, shape, and homogeneity of pores in the gel network, which was related to the WHC of the EWPG. In this study, the porosity in the EWPG increased with the increasing inlet temperature and achieved a minimum value of 50.07% at 140 °C. And the porosity reached a maximum of 56.78% at 900 mL/h. It was known that the formation of protein gel was determined by the rate of protein denaturation and aggregation. When the denaturation rate exceeded the aggregation rate, a dense and uniform gel structure would be produced [46]. The microstructure showed that a uniform gel network was formed at an inlet temperature of 140 °C, and the porosity was larger at a low flow rate. The gel network structure with large pores was linked to weakened rheological behavior and a reduced WHC.

D_f_ always represented the complexity of the gel network and the diversity of aggregates and was applied to describe the generation of aggregates during thermal processing. A low value of D_f_ suggested a more homogeneous gel structure and a lower degree of fractal self-similarity [17]. The maximum D_f_ of the EWPG was 2.745 at an inlet temperature of 140 °C. This was due to the fact that a higher inlet temperature was beneficial for the unfolding of the molecular structure of EWP, and the content of soluble aggregates increased, which was conducive to the formation of a homogeneous linear gel network [47]. However, as the inlet temperature was too high, insoluble aggregates were produced, which impeded the formation of the gel network. The degree of fractal self-similarity of the EWPG declined with a reduced flow rate, with a minimum value of D_f_ (2.709) at 720 mL/h.

## 4. Conclusions

In this study, the effects of two critical spray-drying conditions (inlet temperature and flow rate) on the physicochemical and structural properties of egg white protein and the gel produced were analyzed in order to provide valuable insights for optimizing the production of egg-based food products using spray-drying. The results suggest that during spray-drying, the exposure of hydrophobic groups in the EWP increased and promoted protein aggregation. The primary intermolecular forces governing EWPG formation were disulfide bonds, followed by hydrophobic interactions and ionic bonds. At an inlet temperature of 140 °C, the EWPG exhibited a denser network structure, with a porosity of 50.07% and a WHC of 70.2%, along with improved hardness and elasticity. However, excessively high inlet temperatures led to the over-aggregation of EWP, resulting in a sheet-like gel network with larger pores. With the flow rate reduced from 1260 to 720 mL/h, the surface hydrophobicity of EWP was enhanced, and the WHC was significantly decreased. Moreover, the low flow rate was beneficial to produce an EWPG with good hardness and chewiness, and the maximum appeared at a flow rate of 900 mL/h. The rheology results suggest that large aggregates formed by pre-aggregation would accelerate the formation of a primary gel network. The EWPG exhibited weak frequency dependence and predominantly elastic behavior. Under large deformation, the EWPG displayed complex intra-cyclic rheological behavior and was subjected to fifth-order nonlinear viscous harmonics at yielding. These findings provide significant insight into the relationship between spray-drying conditions and the properties of egg white protein and its gel, especially for LAOS analysis, which is useful in actual applications. Hence, this work provides optimization strategies in the drying process and is beneficial for the designation of actual egg-based products with desirable functional characteristics.

## Figures and Tables

**Table 1:** Spray-drying conditions of different samples.

	Inlet Temperature	Flow Rate	Spray Pressure
100 °C-EWP	100 °C	1260 mL/h	0.2 MPa
120 °C-EWP	120 °C	1260 mL/h	
140 °C-EWP	140 °C	1260 mL/h	
160 °C-EWP	160 °C	1260 mL/h	
1260 mL/h-EWP	140 °C	1260 mL/h	
1080 mL/h-EWP	140 °C	1080 mL/h	
900 mL/h-EWP	140 °C	900 mL/h	
720 mL/h-EWP	140 °C	720 mL/h	

**Table 2:** Solubility and pH of EWP treated with different inlet temperatures and flow rates.

Parameter	Solubility (g/100 g)	Moisture Content	pH
100 °C	93.40 ± 1.16 ^d^	9.98% ± 0.13% ^a^	10.26 ± 0.02 ^e^
120 °C	94.79 ± 1.21 ^bc^	9.11% ± 0.10% ^b^	10.27 ± 0.05 ^e^
140 °C	95.40 ± 0.18 ^ab^	8.44% ± 0.10% ^c^	10.42 ± 0.02 ^c^
160 °C	92.99 ± 0.46 ^d^	6.39% ± 0.18% ^d^	10.36 ± 0.02 ^d^
1260 mL/h	95.33 ± 0.87 ^ab^	8.54% ± 0.03% ^c^	10.37 ± 0.03 ^d^
1080 mL/h	96.49 ± 0.61 ^a^	6.05% ± 0.21% ^e^	10.50 ± 0.01 ^b^
900 mL/h	96.51 ± 0.82 ^a^	5.62% ± 0.23% ^f^	10.37 ± 0.02 ^d^
720 mL/h	93.74 ± 0.20 ^cd^	5.09% ± 0.17% ^g^	10.55 ± 0.03 ^a^

Note: The results are presented as means with standard deviation. Means with different lowercase letters are significantly different (*p* < 0.05).

**Table 3:** Power-law model parameters of frequency sweep curves of EWPG treated with different inlet temperatures and flow rates.

Parameter	Storage Modulus (G′)			Storage Modulus (G″)		
	K′	n′	Adj. R^2^	K″	n″	Adj. R^2^
100 °C	3484.10 ± 2.98	0.04	0.998	219.50 ± 2.02	0.08	0.960
120 °C	2781.29 ± 2.43	0.04	0.998	176.48 ± 1.70	0.09	0.962
140 °C	4119.36 ± 5.57	0.04	0.994	393.24 ± 4.12	0.06	0.874
160 °C	3348.80 ± 6.07	0.04	0.993	243.50 ± 2.72	0.08	0.942
1260 mL/h	3838.23 ± 9.20	0.04	0.988	260.68 ± 3.87	0.08	0.894
1080 mL/h	3589.93 ± 5.14	0.03	0.988	198.06 ± 2.02	0.07	0.925
900 mL/h	4321.64 ± 0.90	0.05	0.999	402.97 ± 2.83	0.08	0.964
720 mL/h	4867.34 ± 31.99	0.02	0.750	347.55 ± 1.77	0.07	0.981

Note: The results are presented as means with standard deviation.

**Table 4:** Parameters of Burger’s model for EWPG treated with different inlet temperatures and flow rates in creep–recovery test.

Parameter	J_max_ × 10^−5^ (Pa^−1^)	J_0_ × 10^−5^ (Pa^−1^)	J_1_ × 10^−5^ (Pa^−1^)	Λ (s)	η_0_ (kPa × s)	R (%)	Adj. R^2^
100 °C	123.57 ± 1.91	88.65 ± 0.17	14.44 ± 1.38	12.11 ± 0.79	14.65 ± 0.52	83.43%	0.991
120 °C	109.02 ± 2.35	75.99 ± 0.20	14.05 ± 0.36	7.92 ± 0.55	15.81 ± 0.60	82.59%	0.988
140 °C	66.29 ± 1.96	42.60 ± 0.16	9.96 ± 0.28	7.35 ± 0.57	21.85 ± 0.89	79.29%	0.985
160 °C	84.24 ± 1.57	58.50 ± 0.12	11.10 ± 0.24	10.84 ± 0.60	20.50 ± 0.66	82.62%	0.993
1260 mL/h	66.34 ± 2.01	42.58 ± 0.25	9.91 ± 0.42	7.10 ± 0.84	21.67 ± 1.32	79.12%	0.966
1080 mL/h	48.61 ± 1.95	28.28 ± 0.24	9.53 ± 0.34	4.65 ± 0.48	27.77 ± 1.75	77.78%	0.965
900 mL/h	38.87 ± 1.20	22.00 ± 0.14	6.59 ± 0.23	6.18 ± 0.60	29.20 ± 1.29	73.55%	0.978
720 mL/h	27.73 ± 1.13	15.16 ± 0.11	5.23 ± 0.17	5.14 ± 0.48	40.90 ± 1.89	73.53%	0.977

Note: The results are presented as means with standard deviation.

## Data Availability

The original contributions presented in this study are included in this article. Further inquiries can be directed to the corresponding author.
